# Thermal Properties of Bayfol^®^ HX200 Photopolymer

**DOI:** 10.3390/ma13235498

**Published:** 2020-12-02

**Authors:** Pierre-Alexandre Blanche, Adoum H. Mahamat, Emmanuel Buoye

**Affiliations:** 1 College of Optical Sciences, University of Arizona, 1630 E. University Blvd, Tucson, AZ 85721, USA; 2 Naval Air Systems Command/NAWCAD, Patuxent River, MD 20670, USA; adoum.mahamat@navy.mil (A.H.M.); emmanuel.buoye1@navy.mil (E.B.)

**Keywords:** photopolymer, temperature, hologram, CTE, thermal degradation, refractive index

## Abstract

Bayfol^®^ HX200 photopolymer is a holographic recording material used in a variety of applications such as a holographic combiner for a heads-up display and augmented reality, dispersive grating for spectrometers, and notch filters for Raman spectroscopy. For these systems, the thermal properties of the holographic material are extremely important to consider since temperature can affect the diffraction efficiency of the hologram as well as its spectral bandwidth and diffraction angle. These thermal variations are a consequence of the distance and geometry change of the diffraction Bragg planes recorded inside the material. Because temperatures can vary by a large margin in industrial applications (e.g., automotive industry standards require withstanding temperature up to $125{\;}^{{}^{\circ}}$C), it is also essential to know at which temperature the material starts to be affected by permanent damage if the temperature is raised too high. Using thermogravimetric analysis, as well as spectral measurement on samples with and without hologram, we measured that the Bayfol^®^ HX200 material does not suffer from any permanent thermal degradation below $160{\;}^{{}^{\circ}}$C. From that point, a further increase in temperature induces a decrease in transmission throughout the entire visible region of the spectrum, leading to a reduced transmission for an original 82% down to 27% (including Fresnel reflection). We measured the refractive index change over the temperature range from $24{\;}^{{}^{\circ}}$C to $100{\;}^{{}^{\circ}}$C. Linear interpolation give a slope $4.5\times 10^{-4}\;K^{-1}$ for unexposed film, with the extrapolated refractive index at $0{\;}^{{}^{\circ}}$C equal to $n_{0}=1.51$. This refractive index change decreases to $3\times 10^{-4}\;K^{-1}$ when the material is fully cured with UV light, with a $0{\;}^{{}^{\circ}}$C refractive index equal to $n_{0}=1.495$. Spectral properties of a reflection hologram recorded at 532 nm was measured from $23{\;}^{{}^{\circ}}$C to $171{\;}^{{}^{\circ}}$C. A consistent 10 nm spectral shift increase was observed for the diffraction peak wavelength when the temperature reaches $171{\;}^{{}^{\circ}}$C. From these spectral measurements, we calculated a coefficient of thermal expansion (CTE) of $384\times 10^{-6}\;K^{-1}$ by using the coupled wave theory in order to determine the increase of the Bragg plane spacing with temperature.

## 1. Introduction

Over the past few decades, most imaging and nonimaging systems have been designed and built using conventional bulky glass- and metal-based optical elements. Those optical elements have been proved to perform well under many conditions, but they are heavy, expensive, and require long lead time to manufacture. In recent years, optical designers started to shift their focus on the design of thin, lightweight, and easy-to-manufacture optical elements using the method of holographic recording [1,2,3]. Typically, holographic optical elements can be recorded on several different photosensitive materials such as dichromated gelatin, silver halide, photoresist, and photopolymer [4].

As the demand for small, lightweight, and compact optical systems has grown since augmented and virtual reality technologies have entered the optics industry, interest in holographic optical elements has also rapidly increased. Holographic optical elements are cheap, easy to manufacture, and sensitive to wavelength and incidence angle; they also provide high diffraction efficiency. These optical elements diffract light through the refractive index modulation obtained through their recording and development processes [5,6].

For many applications, the thermal response of the holographic material is particularly important. The linear coefficient of thermal expansion (CTE) can have an effect on both the diffraction spectrum and diffraction angle of the hologram, changing the color and modifying the direction of the diffracted beam [7].

In the case of a holographic optical element such as a lens, the temperature can change the focal length [8]. In the case of dispersion gratings or holographic notch filters, there can be a shift in the wavelength distribution that affects their use in spectrometry or in a laser cavity [9,10,11]. For a grating coupler such as those used in holographic combiners for augmented reality and for a heads-up display, this could modify both the color and the field of view of the system [12,13,14].

It is also important to consider the thermal stability of the holographic material for integration into industrial processes and applications [15]. The holographic material and the diffractive structure contained within should be able to withstand the high temperatures encountered during thermoplastic molding and extrusion, multilayers lamination (such as in windshield and security windows), and their use in extreme environments such as defined in automotive, military, and aerospace specifications (up to $+125{\;}^{{}^{\circ}}$C) [16,17].

Volume holograms recorded in acrylamide-based photopolymers were investigated for their operational range and reversibility over temperature range of 15–$50{\;}^{{}^{\circ}}$C, and relative humidity of 10–80% [18,19,20]. This material experiences a red wavelength shift in its diffraction response with increased temperature and humidity. Liu et al. [21] investigated the spectral properties of the DCG-based holograms under different temperatures and humidity conditions. They found that the peak diffracted wavelength decreased with temperature and thermal processing time. Lin et al. [22] studied the temperature effect in PQ:PMMA photopolymer and reported enhancement of the diffraction efficiency with temperature postprocessing. SU-8, a commercial photoresin used for photolithography and surface relief holographic gratings, has been shown to shrink, soften, and even collapse at temperatures just above $100{\;}^{{}^{\circ}}$C. [23]. However, to our knowledge, no one has studied the thermal response of the Bayfol^®^ HX200 photopolymer material.

Bayfol^®^ HX200 photopolymer is a holographic recording material distributed by Covestro that has found uses in many applications such as the heads-up display, augmented reality glasses, solar concentrator, and disperser for a spectrometer [14,24,25]. Bayfol^®^ HX200 is an acrylate-based 2-chemistry photopolymer material; its composition and chemistry are described in detail in [26]. The chemical and physical properties of this material, such as the photo-initiation process, spectral photosensitivity, refractive index modulation, and bleaching, have been characterized [26,27,28]. The Bayfol^®^ HX200 film has an average refractive index of $n_{o}=1.49$ and a thickness of d=16 μm, and it can provide a maximum refractive index modulation of Δn=0.03, per the manufacturer’s specifications [29].

Unfortunately, little is known about its thermal response. The only available information, to our knowledge, is the fact that the Bayfol^®^ HX200 is able to withstand the injection molding procedure with a mold temperature of $70{\;}^{{}^{\circ}}$C [30].

In this paper, we present a study of several thermal characteristics of the Bayfol^®^ HX200 that are critical for its use as an holographic material in industrial processes. We measured the thermal degradation using both thermogravimetry and the transmission spectrum. Both techniques are complementary, giving the maximum temperature under which the material should be kept to ensure its proper optical operation. We quantified the variation of the refractive index as a function of temperature between $24{\;}^{{}^{\circ}}$C and $100{\;}^{{}^{\circ}}$C, for unexposed as well as for exposed materials. Knowing the refractive index allows determination of the precise optical path of the light rays inside the sample and permits the accurate optical design of systems that include this material. This is particularly important for holographic waveguides that rely on total internal reflection, which is determined by the refractive index. Finally, we are also reporting the measurement of the CTE of the material calculated from the wavelength drift experienced by a reflection hologram with temperature. The CTE and the refractive index are absolutely necessary parameters to calculate the thermal behavior of an optical system that use the Bayfol^®^ HX200 material. Together, these two parameters determine the change in spectral and angular dispersion of holographic optical elements according to temperature, and they were not known before this study.

## 2. Materials and Methods

### 2.1. Thermal Degradation

To measure the thermal stability of the Bayfol^®^ HX200 material (manufactured by Covestro AG, Leverkusen, Germany), we used both thermogravimetric analysis and spectroscopy.

The thermogravimetric analysis was performed on a TA Instruments (New Castle, DE, USA) TGA 550 machine at a rate of $10{\;}^{{}^{\circ}}$C/min. The photopolymer sample was separated from the backing film and both compounds were measured separately.

To make sure the material does not suffer any optical damage at temperatures below the weight loss recorded by the thermogravimetric experiment, we recorded the transmission spectrum depending on temperature. For this experiment, the sample was prepared as follows: a $4\times 4\;{\mathrm{cm}}^{2}$ piece of Bayfol^®^ HX200 material was laminated on a float glass and exposed to sunlight for 5 min until fully bleached. The material was then encapsulated with another float glass using UV curing optical glue NOA61, which can withstand a temperature of $260{\;}^{{}^{\circ}}$C for three hours according to the manufacturer.

The sample was mounted parallel in front of a first surface aluminum mirror and placed into a temperature-regulated oven. The front side of the oven had a transparent window so that the sample could be illuminated. We used an Oceanoptics (now Ocean Insight, Orlando, FL, USA) USB-4000 fiber fed UV-VIS spectrometer to measure the transmission spectrum of the sample in a double-pass experiment where the back mirror reflected the light back at the spetrometer input fiber. The light source was a halogen lamp that was projected on a diffuser, passing through an aperture, and collimated. The amplitude of the spectrum was calibrated using a dark measurement where the light source was turned off, and a 100% measurement where the sample was removed from the optical path.

A thermocouple was used to measure the temperature at the sample location. The temperature was gradually increased from ambient $23{\;}^{{}^{\circ}}$C to $260{\;}^{{}^{\circ}}$C at a rate of $60{\;}^{{}^{\circ}}C/h$ in incremental steps. The transmission spectrum was acquired at each step once the temperature reached equilibrium. Then, the temperature was increased to the next step. Since the temperatures recorded at the oven controller and at the thermocouple were different, the temperature steps appeared uneven. The reported temperatures were those measured at the thermocouple, which has a greater precision ($0.1{\;}^{{}^{\circ}}$C) than the oven controller. A final spectrum was acquired after the sample was left for 12 h in the oven at $260{\;}^{{}^{\circ}}$C.

### 2.2. Measurement of the Refractive Index Variation with Temperature and Exposure

Holographic optical elements are recorded by interference of two mutually coherent beams within the Bayfol^®^ HX200 photopolymer material. As a result of the interference, a refractive index modulation is created within the active region of the material [5,6]. The refractive index spatial modulation $n_{g}\left(x\right)$, within the active region of the grating is approximated to have a sinusoidal structure and it is defined by Equation (1),

(1) $$n_{g}\left(x\right)=n_{o}+\Delta n_{g}\cos\left(\frac{2\pi x}{\Lambda }\right)$$

where $n_{o}$ is the average refractive index of the cured Bayfol^®^ HX200 photopolymer, $\Delta n_{g}$ is the refractive index modulation created by the interference between the signal and reference beams, and Λ is the period of the refractive index modulation.

Although both the average refractive index and refractive index modulation are assumed to be fixed after recording and processing, it is expected that they might change as a result of heat or refrigeration.

The refractive index measurement was performed using a Metricon 2010 Model (Pennington, NJ, USA). This system consists of a red laser at 637 nm, high refractive index prism, pneumatically operated coupling head, and photodetector. The prism and coupling head are heated to temperature values comprised between $24{\;}^{{}^{\circ}}$C and $100{\;}^{{}^{\circ}}$C with increments of $5{\;}^{{}^{\circ}}$C or less.

**Figure 1:**
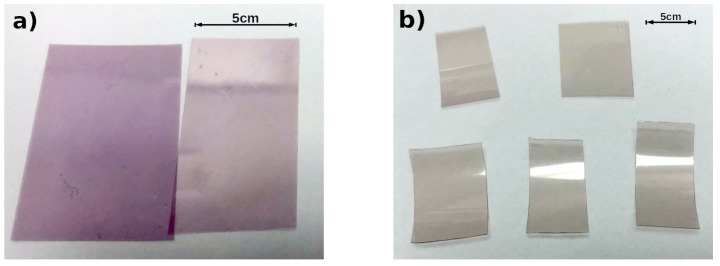
Pictures of Bayfol^®^ HX200 film samples for (**a**) unrecorded/unexposed and (**b**) cured samples.

In Figure 1a, images of the fresh unexposed Bayfol^®^ HX200 material are shown; in Figure 1b, pictures of the material after being fully cured by UV exposure are presented. The sample size was about $5\times 10\;{\mathrm{cm}}^{2}$. The purple coloration of the fresh unexposed samples presented in Figure 1a was due to the photoinitiator that was responsible for the polymerization of the monomer under light exposure. The Bayfol^®^ HX200 material uses a two-component system composed of a dye and an organoborate salt [26]. Once the material has been cured under UV light, the photoinitiator dye is left in a bleached state that is transparent to the visible light. Consequently, the cured samples presented in Figure 1b are colorless. Measurements on the unexposed material were performed with the room light off to avoid any photochemical reaction.

The Bayfol^®^ HX200 photopolymer film was placed on the base of the high refractive index prism and the coupling head was brought pneumatically to ensure that the film was in near contact with the prism. Due to the soft nature of the Bayfol^®^ HX200 photopolymer material, and the fact that any excessive pressure could alter the bonding of its chemical composition, the pneumatic air pressure was set to 20 psi. A piece of electrical tape was placed over the coupling head to minimize stress in the sample under test. For each measurement, the thermocouple controller was set to the desired temperature, and allowed enough time to heat up the sample prior to taking measurement.

For each refractive index versus temperature measurement, ten measurements were collected from different locations within a piece of the film. The mean of the data was then recorded as the average refractive index, $n_{o}$. The temperature of the prism and the coupling were changed to various values ranging between $24^{{}^{\circ}}$ and $100{\;}^{{}^{\circ}}$C with increments of $5{\;}^{{}^{\circ}}$C or less, and a linear function was fitted for all the collected refractive indices.

Extrapolation of the fitted function to an input temperature of $0{\;}^{{}^{\circ}}$C yields the average refractive index at $0{\;}^{{}^{\circ}}$C. The slope of the line indicates the variation of refractive index with temperature, $\frac{\partial n}{\partial T}$. The linear fit function can be expressed as

(2) $$n_{0}=\frac{\partial n}{\partial T}T+n\left[0\right].$$

Since the holographic recording is usually done at room temperature, the refractive index modulation is calculated as the difference between the values of $n_{0}$ for unexposed/unrecorded film and cured film. The cured sample was laminated over 3 mm thick BK7 substrate glass (Thorlabs Inc., Newton, NJ, USA) and placed under 275W Xenon lamp (Osram HLX 64656-FNT Xenophot, Osram Sylvania, Wilmington, MA, USA) for fourteen hours, followed by one hour exposure under the sun to make sure the sample was fully cured.

### 2.3. Linear Coefficient of Thermal Expansion

For volume phase holographic gratings such as the one recorded in Bayfol^®^ HX200, the energy distribution depending on the angle and wavelength can be calculated using electromagnetic propagation theory or coupled wave analysis [5]. However, the direction ($\theta_{B}$) and wavelength ($\lambda_{B}$) of the maximum efficiency can be predicted simply by using Bragg’s equation:

(3) $$sin\left(\theta_{B}\right)=\frac{\lambda_{B}}{2n\Lambda }$$

where *n* is the refractive index of the material and Λ is the distance between the modulation planes (Bragg planes).

When the material in which the hologram is recorded shrinks or swells, the spacing and angle of the modulation planes are affected, changing the $\vec{K}$ vector of the grating (with |K|=2π/Λ). This impacts the diffraction angle and the diffracted spectrum of the hologram.

In the simplified configuration of a transmission hologram with no slant angle, only the lateral spacing of the modulation planes changes ($|K^{\prime }|=2\pi /\left(\Lambda +\Delta \Lambda \right)$), which influences the diffraction angle for a specific wavelength:

(4) $$sin\left(\theta_{B}^{\prime }\right)=sin\left(\theta_{B}\right)\frac{1}{1+\frac{\Delta \Lambda }{\Lambda_{B}}}$$

Usually, this effect is not particularly visible since the material is laterally constrained by the substrate on which it is laminated. In the case where the two materials (hologram and substrate) have different coefficients of thermal expansion, the system is bending, introducing even more aberrations that could not easily be separated.

On the other hand, for a reflection grating with its modulation planes parallel to the substrate, a change of the material in the thickness direction affects the diffracted wavelength, keeping the diffraction angle the same:

(5) $$\lambda_{B}^{\prime }=\lambda_{B}\left(1+\frac{\Delta \Lambda }{\Lambda_{B}}\right)$$

This wavelength change can easily be detected by a spectrometer and is not coupled to any other effect, making it a good candidate for the measurement of the material CTE.

In the more general case where the modulation planes have a slant angle different from 0 or π/2, a more rigorous calculation, such as coupled wave analysis, is required to determine the perturbation on both diffraction angle and wavelength.

It should also be noted that the wavelength shift in reflection hologram due to the material swelling has been extensively studied in other materials such as silver halide or dichromated gelatin [31]. This effect has been used for tuning the hologram color and for the production of three colors (red, green, and blue) 3D images with a single laser [32]. However, in the case of these collagen-based materials, the swelling is due to the absorption of water rather than because of CTE, and the effect is orders of magnitude larger than what is expected with temperature change.

For testing the temperature dependence on the spectrum of a reflection hologram, the Bayfol^®^ HX200 sample was prepared as in the previous section: a $3\times 6\;{\mathrm{cm}}^{2}$ sample was laminated on float glass, then encapsulated after the hologram was recorded. The reflection hologram was recorded with a 532 nm laser with one beam orthogonal to the sample and the other beam incident at $5^{{}^{\circ}}$ angle. This angle was introduced so the diffracted beam could be easily separated from the front face reflection.

**Figure 2:**
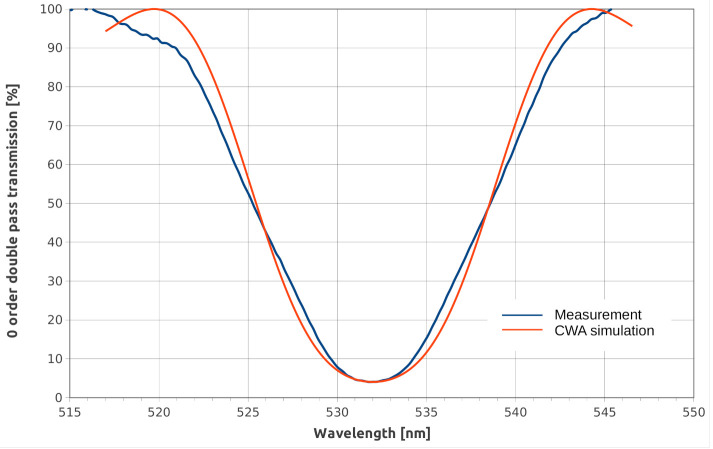
Double-pass transmission spectrum (zero-order) of the reflection hologram interpolated by coupled wave analysis.

The transmission spectrum (zero-order) was measured in a double-pass experiment where the illumination light was reflected back by a mirror located behind the sample. The orientation of the incident illumination and the mirror angle were optimized to achieve maximum diffraction efficiency at 532 nm. In Figure 2, the interpolation of the spectrum measured at room temperature with coupled wave analysis is presented. The best fit parameters were Λ=5637 lp/mm (532 nm wavelength with $5^{{}^{\circ}}$ and $180^{{}^{\circ}}$ (=$0^{{}^{\circ}}$ opposite direction) incidence angles), 7.2 μm effective thickness, and a refractive index modulation of Δn=0.026.

The temperature was gradually raised from ambient to $170{\;}^{{}^{\circ}}$C over the course of 3 h, during which spectra were acquired at regular intervals. The sample was then allowed to cool down back to ambient temperature over 12 h. Finally, another temperature cycle was run with more spectra acquisition.

## 3. Results and Discussion

### 3.1. Thermal Degradation

**Figure 3:**
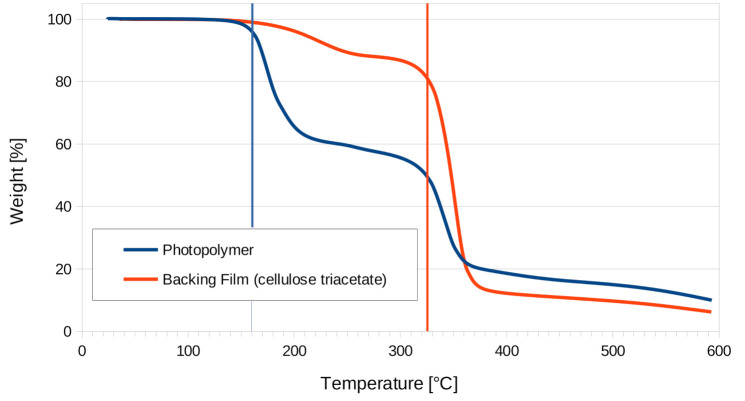
Thermogravimetric analysis of the Bayfol^®^ HX200 photopolymer and the cellulose triacetate backing.

The thermogravimetric analysis of both the Bayfol^®^ HX200 photopolymer material and its cellulose triacetate backing (without photopolymer) is presented in Figure 3.

The photopolymer followed a multistage decomposition that started at $160{\;}^{{}^{\circ}}$C (first onset) and reached its midpoint at $180{\;}^{{}^{\circ}}$C. The backing film showed an initial degradation at the same temperature that is believed to be due to some photopolymer residue on our sample. A much larger weight loss appeared at $325{\;}^{{}^{\circ}}$C, which is consistent with the literature on cellulose triacetate [33].

### 3.2. Spectral Measurement

**Figure 4:**
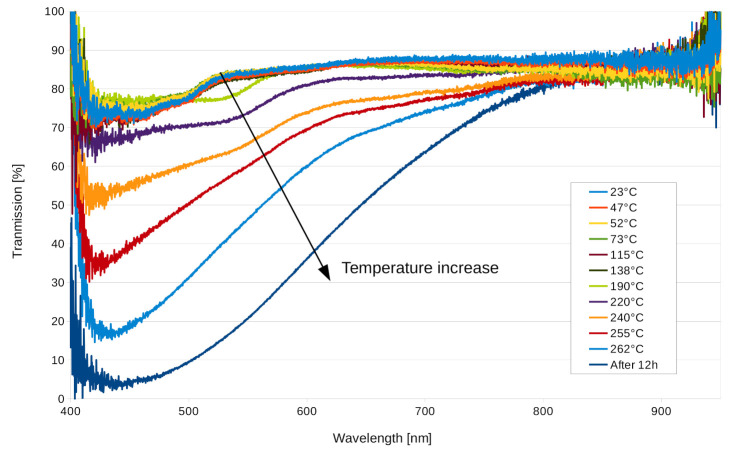
Transmission spectrum of the Bayfol^®^ HX200 sample without hologram, depending on temperature.

**Figure 5:**
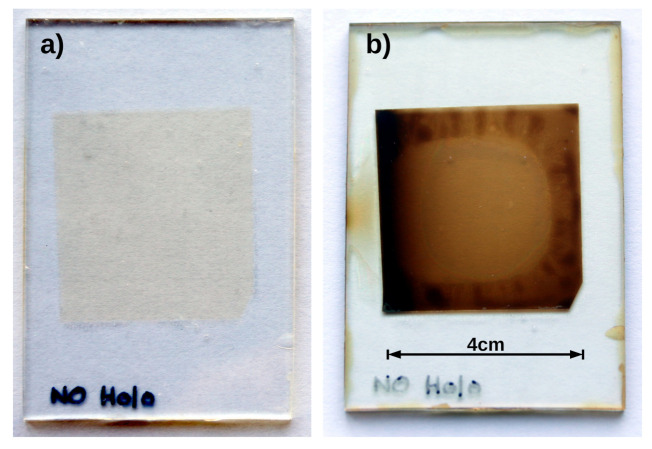
Pictures of the sample before thermal treatment (**a**), and after 12 h at $262{\;}^{{}^{\circ}}$C (**b**).

In Figure 4, the results of the spectral measurement depending on temperature are presented. The initial spectrum taken at $47{\;}^{{}^{\circ}}$C shows the residual absorption of the sample composed of glass, Bayfol^®^ HX200, and NOA60 glue, as well as the Fresnel reflection from the different interfaces. It can be noted from the picture taken of the sample before thermal treatment (Figure 5a) that the Bayfol^®^ HX200 material is slightly yellow. No spectral change was observed up to $138{\;}^{{}^{\circ}}$C. The next spectrum recorded at $190{\;}^{{}^{\circ}}$C shows a decrease in transmission around 530 nm consistent with the thermogravimetric measurement. This indicates that the decomposition temperature was reached. As the temperature kept increasing, the transmission kept decreasing over the entire 400 nm to 840 nm band.

Pictures of the sample before and after thermal treatment are presented in Figure 5. It can be noticed from Figure 5b that the brown coloration of the sample was only due to the Bayfol^®^ HX200 material, since there was no color change for the NOA61 optical glue around the material. The optical transmittance of the sample before thermal treatment was 82% integrated over the entire visible spectrum (450–750 nm) and included the Fresnel reflection. After the thermal treatment ($262{\;}^{{}^{\circ}}$C for 12 h), the optical transmittance was reduced to 27%.

The precise chemical reaction responsible for the darkening of the photopolymer with temperature is beyond the scope of this research, but it can be deduced from similar behaviors observed in other polymers that the material undergoes thermal oxidation and further breakdown of the polymer chains [34]. To avoid these irreversible reactions, the Bayfol^®^ HX200 material should be kept below $160{\;}^{{}^{\circ}}$C at all times.

### 3.3. Refractive Index

**Figure 6:**
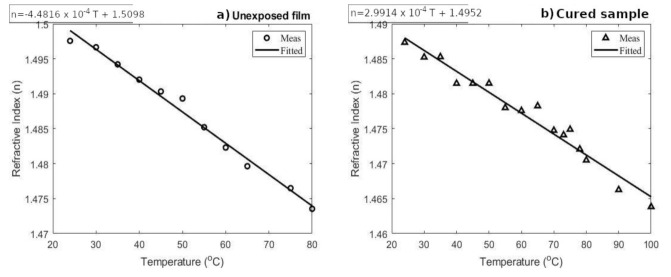
Refractive index variation depending on temperature for the (**a**) unexposed/unrecorded samples and (**b**) cured sample.

Figure 6 shows the change of the refractive index of the Bayfol^®^ HX200 samples as they were heated to temperatures between $24{\;}^{{}^{\circ}}$C and $100{\;}^{{}^{\circ}}$C. The plots show that increased temperatures caused the refractive index to drop for both samples. The highest change is seen with the unrecorded samples as $\frac{\partial n}{\partial T}$ is 4.5 × $10^{-4}\;K^{-1}$, when the cured samples have the value 3 × $10^{-4}\;K^{-1}$.

From the values of the refractive index presented in Figure 6, it can be noted that the refractive index change due to material curing by light exposure was around 0.015 for the cured sample. This Δn did not reach the maximum of 0.03 as indicated by the Bayfol^®^ HX200 manufacturer data sheet [35]. Knowing the fact that the photopolymer materials rely on the photoinitiated cross-linking, monomer diffusion, and further polymerization to achieve high refractive index modulation [4], it can be understood that uniform illumination as used in this study does not allow for maximum refractive index change. In the case of large area illumination, the monomers could not diffuse far enough from the unexposed regions of the material to increase the material density and refractive index.

### 3.4. Linear Coefficient of Thermal Expansion

**Figure 7:**
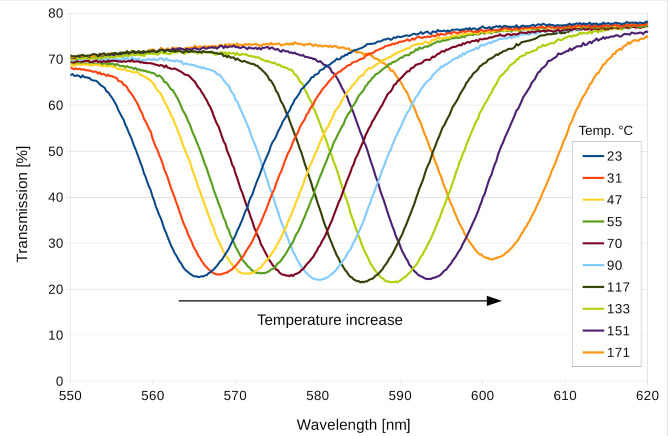
Transmission spectrum of the Bayfol^®^ HX200 reflection hologram depending on temperature.

Figure 7 shows how the transmission spectrum of the reflection hologram drifted with temperature. It can be seen that from $23{\;}^{{}^{\circ}}$C to $171{\;}^{{}^{\circ}}$C, the transmission minimum drifted from 565 nm to 602 nm.

**Figure 8:**
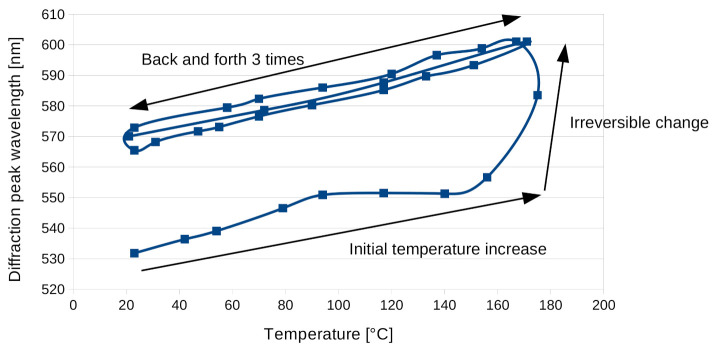
Wavelength minimum of the transmission spectra of the Bayfol^®^ HX200 reflection hologram depending on temperature for two temperature cycles.

**Figure 9:**
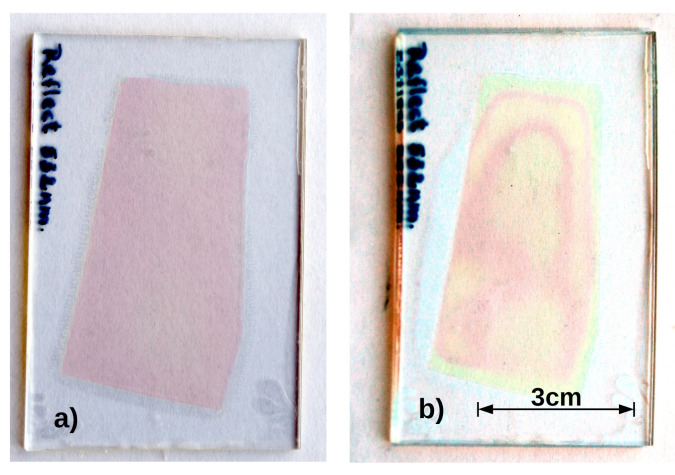
Pictures of the reflection hologram sample before thermal treatment (**a**) and after initial temperature increase from ambient temperature to $170{\;}^{{}^{\circ}}$C (**b**). Note that the pink coloration of the sample was not due to the material absorption but to the diffraction of the green wavelength that was no more present in the transmission spectra (white light − green = pink).

In Figure 8, the wavelength of the transmission minimum was plotted depending on temperature for two temperature cycles (back and forth) between $23{\;}^{{}^{\circ}}$C and $170{\;}^{{}^{\circ}}$C. During the initial temperature increase, an irreversible change occurred at $170{\;}^{{}^{\circ}}$C with a sudden increase in the minimum wavelength. After observation of the sample, we noticed that the NOA glue has softened and allowed the sample to shift from the cover glass. This shift induced a color change in the reflection spectrum of the hologram that can be seen in Figure 9b.

Figure 9 shows pictures of the reflection hologram sample before thermal treatment (left) and after the initial temperature increase to $170{\;}^{{}^{\circ}}$C (right). One can see some permanent change in the form of nonuniform color (pink or yellow) of the hologram after the thermal treatment. Note that the coloration of the sample was not due to material absorption, but to the diffraction from the hologram. It has to be noted that after this initial irreversible change, further temperature variation from $20{\;}^{{}^{\circ}}$C to $170{\;}^{{}^{\circ}}$C induces a linear and fully reversible change in diffracted wavelength.

**Figure 10:**
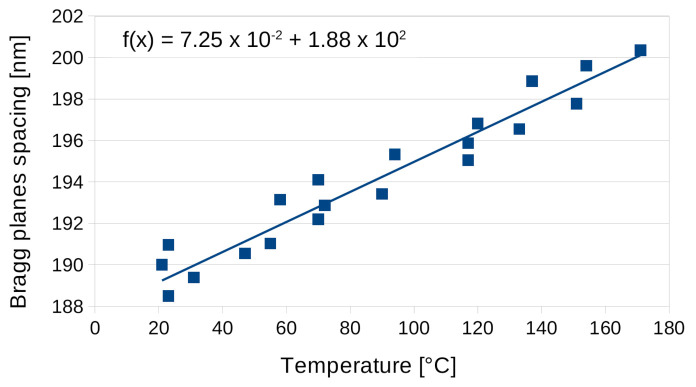
Bragg planes spacing for the Bayfol^®^ HX200 reflection hologram depending on temperature for 1.5 temperature cycles.

To calculate the linear coefficient of thermal expansion (CTE) for the Bayfol^®^ HX200 material, we used Equation (5) to retrieve the spacing of the Bragg planes spacing from the measurement of the diffraction peak minimum wavelength as a function of temperature. This spacing parameter is presented in Figure 10 where it is interpolated by a linear regression. The origin of the straight line interpolation gives the original distance between the planes (*L*), and the slope gives the distance increase depending on temperature (ΔL/ΔT). The CTE is defined as:

(6) $$\alpha =\frac{\Delta L}{L.\Delta T}=384\times 10^{-6}\;K^{-1}$$

The CTE calculated value $384\times 10^{-6}\;K^{-1}$ for the Bayfol^®^ HX200 is of the same order of magnitude as the literature values for other polymeric materials such as polypropylene $150\times 10^{-6}\;K^{-1}$, polystyrene $150\times 10^{-6}\;K^{-1}$, or teflon $\;175\times 10^{-6}\;K^{-1}$ [36,37,38].

## 4. Conclusions

Thermogravimetric measurements show that the Bayfol^®^ HX200 material does not suffer from any thermal degradation before $160{\;}^{{}^{\circ}}$C. Up to that temperature, holograms recorded in this material experience only reversible change, expressed as thermal dilatation between the Bragg planes. Irreversible change in the hologram diffraction spectrum started at $170{\;}^{{}^{\circ}}$C, and irreversible change in the transmission spectrum of the material was recorded starting at $190{\;}^{{}^{\circ}}$C.

Refractive index variation depending on the temperature was measured as 4.5×$10^{-4}\;K^{-1}$ for the unexposed material and 3.3×$10^{-4}\;K^{-1}$ once the material was fully cured. These changes are linear depending on the temperature over a range from $24{\;}^{{}^{\circ}}$C to $100{\;}^{{}^{\circ}}$C.

A CTE of $384\times 10^{-6}\;K^{-1}$ was calculated by measuring the spectral shift of a reflection hologram depending on temperature. This color shift was due to the dilatation between the Bragg planes experienced when the photopolymer material swelled under temperature increase.

These results will be valuable to predict the characteristics of holographic optical elements and other holograms in applications and processes where thermal variation is expected.
